# A case of concurrent Alport syndrome and Nail-patella syndrome posing diagnostic challenge without genetic testing

**DOI:** 10.1186/s12882-025-04606-1

**Published:** 2025-11-26

**Authors:** Winston Wing-Shing Fung, Maggie Lo-Yee Yau, Pensi Ping Hei Lam, Cheuk-Chun Szeto, Shuk-Ching Chong, Kai-Ming Chow

**Affiliations:** 1 Division of Nephrology, Department of Medicine & Therapeutics, Prince of Wales Hospital, Hong Kong, Hong Kong China; 2 Department of Paediatrics & Adolescent Medicine, Prince of Wales Hospital, Hong Kong, Hong Kong China; 3 Department of Anatomical & Cellular Pathology, Prince of Wales Hospital, Hong Kong, Hong Kong China; 4 Li Ka Shing Institute of Health Sciences (LiHS), The Chinese University of Hong Kong, Hong Kong, Hong Kong China; 5 Joint Baylor College of Medicine-CUHK Centre of Medical Genetics, The Chinese University of Hong Kong, Hong Kong, Hong Kong China; 6 Department of Medicine & Therapeutics, Prince of Wales Hospital, The Chinese University of Hong Kong, Shatin, NT, Hong Kong China

**Keywords:** Alport syndrome, Nail-patella syndrome, Hereditary glomerular basement membrane disease, Genetics

## Abstract

Hereditary glomerular basement membrane disease is a group of conditions caused by genetic mutations in the development and maintenance of the glomerular basement membrane. Alport syndrome is a classic example caused by variants in the genes *COL4A3*, *COL4A4*, or *COL4A5*. Less common example includes nail-patella syndrome (*LMX1B*-associated nephropathy), which is caused by variants in the *LMX1B* gene. The manifestations of *LMX1B*-associated nephropathy and Alport syndrome can overlap because they share abnormalities in type IV collagen, and this can sometimes cause diagnostic challenges. We describe a case of focal segmental glomerulosclerosis with the genetic test revealing concurrent variants of both Alport syndrome and nail-patella syndrome after noting features of nail-patella syndrome on clinical examination, although the kidney biopsy showed features compatible with Alport syndrome. Our case highlighted the importance of astute clinical examination backed up by genetic testing, which aids in diagnosis and subsequent management.

## Introduction

Hereditary glomerular basement membrane (GBM) disease is a group of conditions caused by genetic mutations in the development and maintenance of the GBM, leading to defects in the glomerular filtration barrier [1]. A well-described hereditary GBM disease is Alport syndrome, which is associated with a progressive glomerular disease, hearing loss, and lens defects due to genetic variants in the genes *COL4A3*, *COL4A4*, or *COL4A5* [2]. Another classical example is thin membrane disease, which is caused by less severe variants in the same collagen proteins [3]. Other less common diseases include Pierson syndrome which has variants in the *laminin β2* (*LAMB2*) gene and nail-patella syndrome (also known as *LMX1B*-associated nephropathy) which has mutations in the *LMX1B* gene [1, 4]. The manifestations of *LMX1B*-associated nephropathy and Alport syndrome can overlap because they share abnormalities in type IV collagen.

While the definitive diagnosis of these diseases is typically achieved through kidney biopsy with electron microscopic examination, genetic testing has revolutionised the diagnostic paradigm as the preferred method, given that it is less invasive and has good diagnostic specificity (e.g. 95% in Alport syndrome) [5]. Both diseases can share similar histological findings, which may cause diagnostic challenges. We describe a case of focal segmental glomerulosclerosis with the eventual genetic test revealing concurrent mutation of genes in both Alport syndrome and nail-patella syndrome.

## Case report

**Fig. 1:**
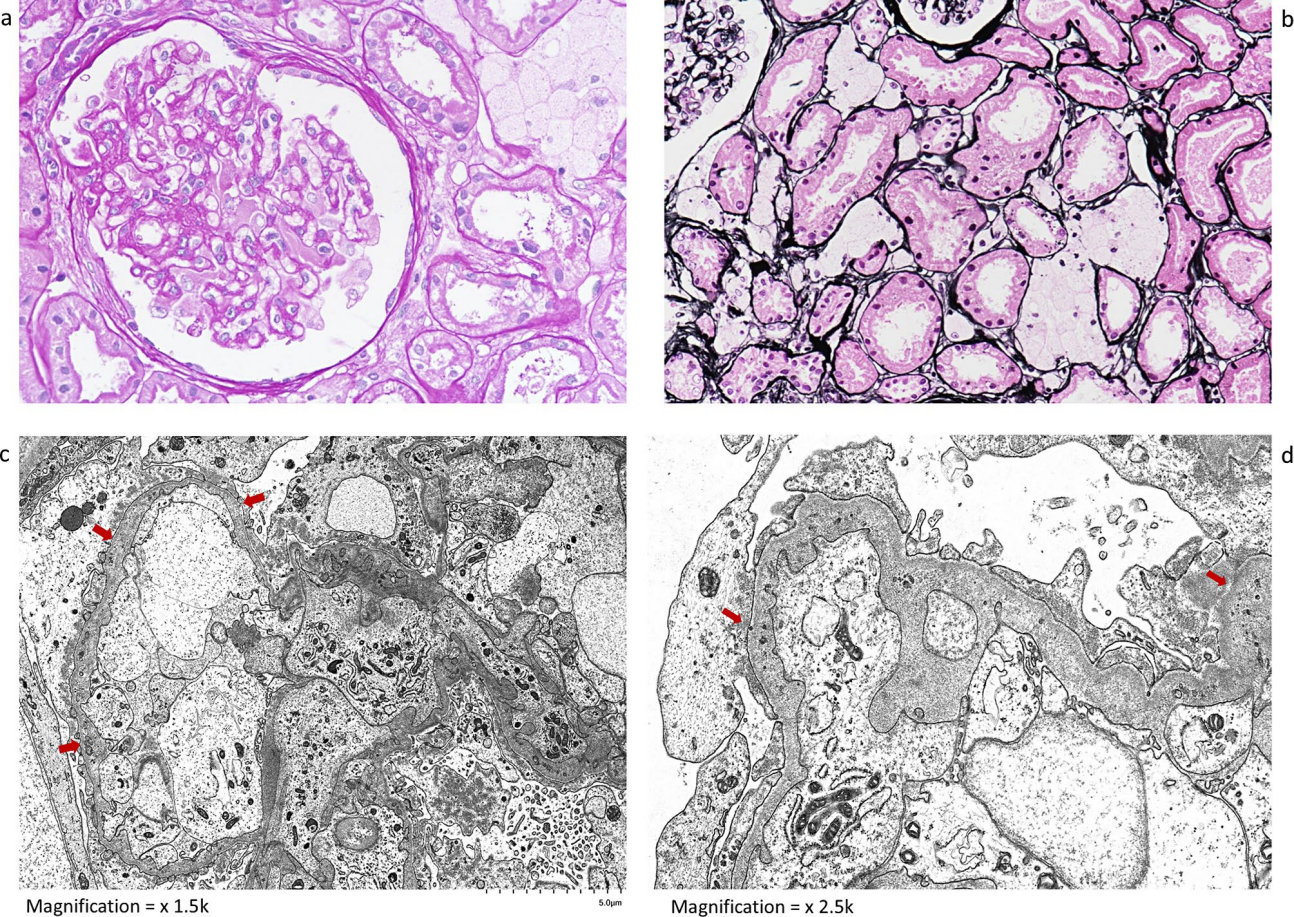
Light microscopy **a**. PAS, **b**. PASM with H&E counterstain, all x 400. Electron microscopy Lead citrate and uranyl acetate, 1c x 1,500; 1d x 2,500. Most glomeruli exhibit minor glomerular abnormality, except for 2 glomeruli with small segmental sclerosis with capsular adhesion, as illustrated here. Two pertinent features are also seen: the presence of interstitial foam cells and the roughened capillary walls (**a** and **b**). Glomeruli show intact capillary lobular architecture, with the absence of electron-dense deposits. The GCBM (glomerular capillary basement membrane) is distinctly irregular due to thinning and thickening (arrows, **c**). The lamina densa is focally disrupted and lamellated, associated with granular fragments or “crumbs” (arrows, Fig. 1**d**)

A 62-years-old woman first presented to us for microscopic haematuria and persistent proteinuria of 0.9 g after her first pregnancy forty years ago at the age of 22 years old. Her kidney function was normal, and the nephritic screening was unremarkable. She underwent a kidney biopsy, which showed minor abnormality except for a small segmental sclerosis in 2 of the 26 glomeruli. Prominent interstitial foam cells are also noted, and tubular atrophy was minimal, accounting for 3% of cortical scarring (Fig. 1). Although arterioles were tortuous, there were no features of hypertension. Immunofluorescence was negative. Electron microscopy subsequently showed an irregular glomerular capillary basement membrane with both thinning and thickening, and foci of disruption and splitting of lamina densa with “crumbs”. There was no “basket weaving” observed (Fig. 1). A diagnosis of Alport syndrome was suggested, and an angiotensin-converting enzyme inhibitor (ACEI) was thus started. There was no ocular involvement, and hearing loss was noted on subsequent examination.

**Fig. 2:**
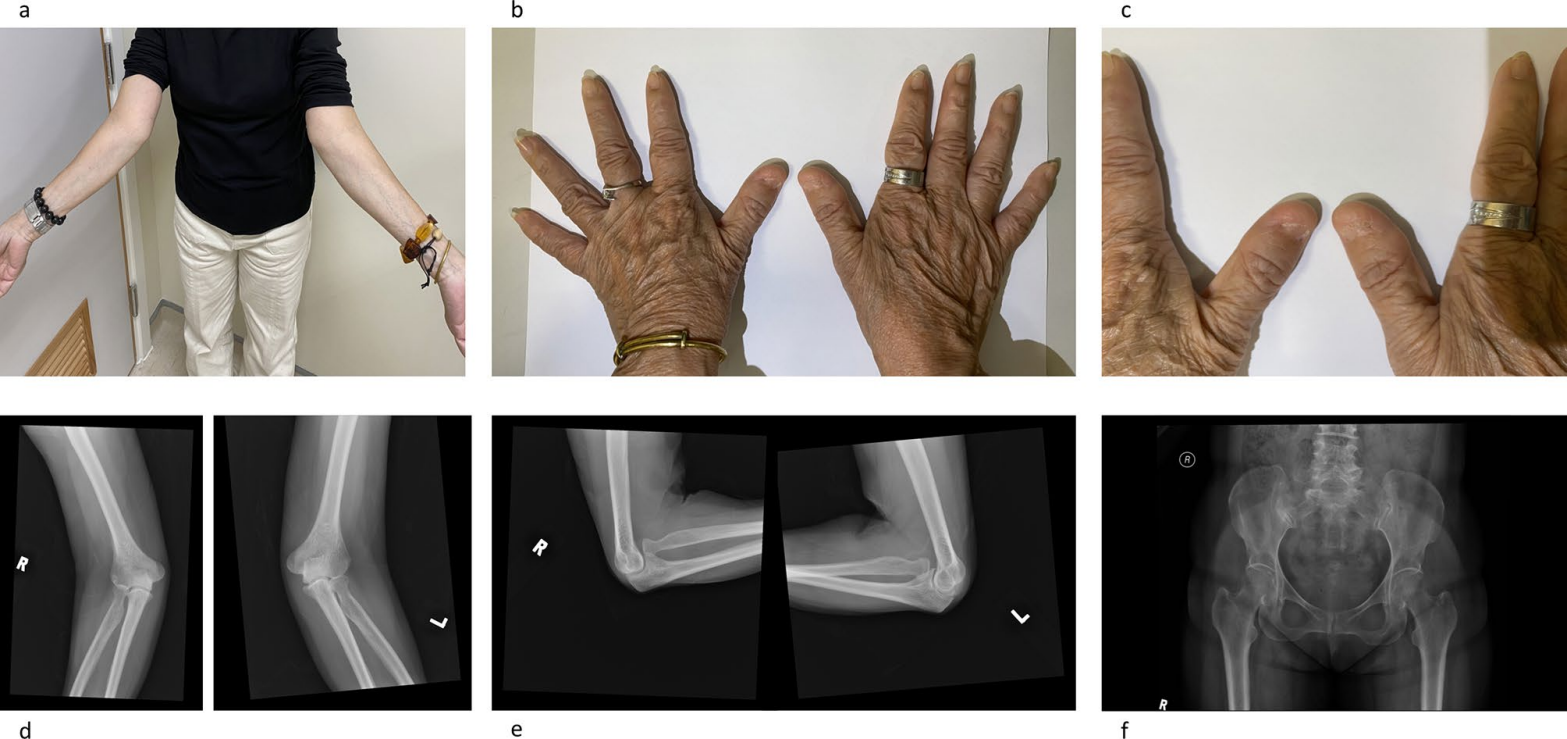
Photograph of the patient showing cubitus valgus (**A**) and dystrophic thumb nails with lack of skin creases over the distal interphalangeal joints of the index fingers (**B**, **C** – close up) as well as elbow X ray with features of nail-patella syndrome (**D**, **E**) and a normal pelvic X ray (**F**)

**Fig. 3:**
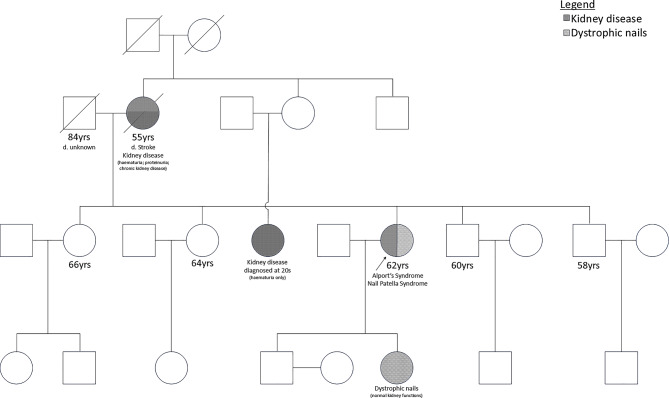
The family pedigree showing several members with kidney dysfunctions as well as dystrophic nails. None of them had any ocular involvement or hearing loss

The patient continued to be followed up in our clinic with stable kidney function. During one of the recent clinic visits when she was seen by our senior physician, she was noted to have dystrophic nails with a lack of skin creases over the distal interphalangeal joints of the index finger and cubitus valgus on clinical examination (Fig. 2). A subsequent elbow X-ray showed hypoplasia of the capitellum with subluxation of the radial heads and mildly abnormal shape of the radial heads bilaterally, appearance in keeping with nail-patella syndrome (Fig. 2). There were no definite posterior iliac horns on the pelvic X-ray. Interestingly, the patient expressed that her daughter also has similar nails and elbow abnormalities. Nail-patella syndrome was thus suspected, and next-generation sequencing of the target genes was performed by oligonucleotide-based target capture (TruSight One Expanded Sequencing Kit) using Illumina NextSeq 2000. The genetic test remarkably confirmed that she has both variants: (1) a heterozygous variant NM_001174147.2:c.691 C >T p. (Arg231Ter) on the *LMX1B* gene and (2) a heterozygous variant NM_000092.5:c.1803 + 2T >C on the *COL4A4* gene. Subsequent analysis showed that the nonsense c.691 C >T variant of *LMXB* is predicted to change the 231st amino acid residue from arginine to a stop codon, and the mRNA is predicted to undergo nonsense-mediated mRNA decay. For the *COL4A4* gene, the c.1803 + 2T >C variant is predicted to disrupt the canonical splice donor site of *COL4A4*, and the mRNA is predicted to undergo nonsense-mediated mRNA decay, of which loss-of-function is a known disease mechanism. Both variants are absent from the control population in gnomAD v3.1.2. Thus, both variants are classified as likely pathogenic (Table 1) according to the ACMG guidelines following discussion in the multi-disciplinary meeting [6, 7]. A more detailed family history was taken, and several family members also showed the presence of haematuria and proteinuria (Fig. 3). She remains well with a stable kidney function while on an ACEI. A cascade screening test is currently underway.

**Table 1:** Variants analysis and interpretation

	Variant	CADD	Polyphen	SIFT	Variant interpretation*
LMX1B: c.691 C > T	Stop-gain variant	41 (pathogenic)	not applicable	not applicable	PVS1 + PM2_moderate + PP5_strong
COL4A4:c.1803 + 2T > C	Splice site variant	30 (pathogenic)	not applicable	not applicable	PVS1 + PM2_mdoerate

Variant analysis in silico tools used: CADD, Combined Annotation Dependent Depletion; Polyphen, Polymorphism Phenotyping; SIFT, Sorting Intolerant From Tolerant. *according to the ACMG guideline

## Discussion

Hereditary glomerular membrane disease is an uncommon but increasingly recognised condition, owing to the improved availability of genetic testing. Indeed, a recent study by Groopman et al. highlighted that genotyping a combined cohort totalling 3315 patients with chronic kidney diseases have yielded genetic diagnosis in about 10% of cases [8]. Interestingly, diagnostic variants were detected across all clinically defined categories, including congenital or cystic kidney disease (127 of 531 patients [23.9%]) and nephropathy of unknown origin (48 of 281 patients [17.1%]). Of the 2187 patients assessed, 34 (1.6%) had genetic findings for medically actionable disorders that, although unrelated to their nephropathy, would also lead to subspecialty referral and inform kidney management [8]. This certainly highlighted the increasing usefulness of genetic testing in accurate diagnosis, which will inform subsequent management.

Alport syndrome is a genetic condition characterized by kidney disease, loss of hearing, and eye abnormalities, due to a mutation in the genes encoding alpha-3, alpha-4, and alpha-5 of type IV collagen (*COL4A3*, *COL4A4*, *COL4A5*) or collagen 4 α345 network [2]. The mode of inheritance can be X-linked (*COL4A5*), autosomal-dominant and autosomal-recessive pattern (*COL4A3* and *COL4A4*), with the most common inheritance being in an X-linked pattern [2]. Historically, kidney biopsy is performed to look for ultrastructural abnormalities such as GBM thinning with the absence of alpha 5 type IV collagen in immunostaining. It is important to note that GBM thinning is a pathological description and it is not pathognomonic nor confirmatory for Alport syndrome. Some subtypes of Alport syndrome can have normal immunostaining, given the presence of residual truncated type IV collagen chain. Genetic testing is now the gold standard investigation, with next-generation sequencing of *COL4A3*, *COL4A4*, and *COL4A5* being recommended in patients with no family history of Alport syndrome [5].

Nail-patella syndrome is a rare disorder with an autosomal dominant inheritance pattern, and it has an incidence rate of one in 50,000 people [4]. It is due to a heterozygous loss-of-function mutation of the *LMX1B* gene on chromosome 9q34. *LMX1B* plays a crucial role in embryonic development and is expressed in various tissues. At least 142 *LMX1B* mutations have been identified so far, as well as evidence of somatic mosaicism, which is known to occur in unaffected parents of patients affected by autosomal dominant disorders [9]. Even though the condition is highly penetrant, there is a high inter- and intrafamilial variability contributing to the condition’s variable clinical manifestations [10]. Typical manifestations include dysplastic nails, absent or hypoplastic patella, elbow dysplasia, and iliac horns. Absence of skin creases over the distal interphalangeal joints, which our patient had (Fig. 2), is also a subtle but characteristic sign of nail-patella syndrome [11]. About 40% of patients may have kidney impairment, and the severity of kidney impairment is the main factor affecting the prognosis and quality of life of patients [12]. The microscopic findings of structural kidney abnormalities in nail-patella syndrome are fairly non-specific, and they include irregular GBM thickening with a “moth-eaten” appearance, due to the deposition of fibrillar collagen-like material, a hallmark of nail-patella syndrome [3].

Our patient is interesting in that she has concurrent variants in both genes and thus has features of both conditions. In retrospect, this case highlights that an astute clinical examination remains irreplaceable to detect subtle signs suggestive of genetic causes. A careful family history may also help to distinguish the subtle features of the two different conditions, which is unfortunately not thoroughly done in reality with an overwhelming clinic. Nonetheless, it remains difficult to distinguish the two underlying causes without genetic testing, even though we have done a kidney biopsy with our cases showing three of the four diagnostic features of Alport syndrome in electron microscopy: thinning and thickening of GBM, splitting of lamina densa, and granular fragments or “crumbs”. Although nail-patella syndrome tends to have irregular GBM thickening, there have been cases of nail-patella syndrome with a novel *LMX1B* variant presented with severe kidney involvement and thin GBM only [13]. Indeed, two further case reports recently showed that *LMX1B*-associated nephropathy can present with Alport syndrome-like phenotype and GBM abnormalities mimicking Alport syndrome [14, 15]. In particular, Ito et al. highlighted the possibility of using low-vacuum scanning electron microscopy for observing and diagnosing *LMX1B*-associated nephropathy [15].

Even though our patient had both mutations, her kidney function remains relatively stable. First, this patient had heterozygous variants in *COL4A4* and not *COL4A5*, which is known to be associated with a worse prognosis, especially in males with X-linked Alport syndrome. Furthermore, the disease severity of nephropathy caused by *LMX1B* nephropathy is diverse, and the genotype-phenotype correlations remain unclear [10, 16]. Less than half of affected individuals with nail-patella syndrome are said to develop nephropathy with proteinuria and haematuria.

We performed a literature search to see whether either of these variants has been reported previously. From the ClinVar database, the variant NM_001174147.2:c.691 C >T on the *LMX1B* gene will result in a premature stop codon (p.Arg231Ter) in exon 4 of the gene within the homeodomain [17]. This will lead to a truncated, nonfunctional protein, which will subsequently disrupt the *LMX1B* protein’s DNA-binding ability, leading to haploinsufficiency. The position and type of this variant are aligned with other similar well-characterised truncating mutations in the homeodomain reported previously [18]. The variant NM_000092.5:c.1803 + 2T >C occurs on intron 23 of the *COL4A4* gene, at the + 2 position of the donor splice site. This disrupts a conserved splice donor site and will likely cause aberrant splicing, leading to a defective or truncated *COL4A4* protein. This specific variant has been previously reported, although available information remained limited [7]. Nevertheless, its mechanism possibly aligns with other well-characterized splice site variants in the *COL4A4* gene [19, 20].

Overall, our case highlighted the importance of astute clinical examination backed up by genetic testing, which aids in the diagnosis and subsequent management. Furthermore, atypical ultrastructural features such as irregular GBM thickening with a “moth-eaten” appearance and podocyte infolding structures within the GBM may be helpful in prompting the possibility of nail-patella syndrome.

## Data Availability

The datasets generated and/or analysed during the current study are available in the ClinVar repository: Accession number SCV006558649 and SCV006558650.
